# Association between metabolic syndrome and lupus nephritis activity

**DOI:** 10.5937/jomb0-45732

**Published:** 2024-06-15

**Authors:** Violeta Rabrenović, Milica Petrović, Milorad Rabrenović, Nemanja Rančić

**Affiliations:** 1 Military Medical Academy, Clinic of Nephrology, Belgrade; 2 University of Defense, Military Medical Academy, Faculty of Medicine, Belgrade; 3 Military Medical Academy, Center for Hyperbaric Medicine, Belgrade; 4 Military Medical Academy, Centre for Clinical Pharmacology, Belgrade

**Keywords:** lupus nephritis, metabolic syndrome, dyslipidemia, activity, lupus nefritis, metabolički sindrom, dislipidemija, aktivnost

## Abstract

**Background:**

Metabolic syndrome (MetS) in patients with systemic lupus erythematosus (SLE) represents an additional burden and a poor prognostic factor for the onset or worsening of atherosclerosis and cardiovascular complications. In many patients with lupus nephritis (LN), MetS is often already manifested initially. Our work aimed to determine the frequency and characteristics of MetS in patients with LN, as well as the relationship components of MetS and characteristics of disease activity.

**Methods:**

The clinical study included 67 patients with LN, 54 (80.59%) female and 13 (19.41%) male, with an average age of 42.86±14.46 years. Patients were divided into two groups: with MetS (35.82%) and without MetS (64.18%), active LN had (34 or 50.74%), and LN in remission (33 or 49.25%). We monitored clinical and biochemical parameters of interest.

**Results:**

Comparing patients with LN collectively, as well as those with MetS and without MetS, we observed that patients with MetS were older (p=0.001), BMI (p<0.001), and systolic arterial pressure was higher (p=0.002), and smokers were more common in this group (p<0.001). In the analysis, increased triglycerides (p<0.001) and creatinine (p=0.027), and decreased albumin (p=0.050) and GFR (p=0.020) were observed in the group with MetS. MetS was present in 44.11% of patients with active LN and in 27.7% with LN in remission. The most common MetS parameter was arterial hypertension (76.6%), which correlated with GFR and creatinine; hypertriglyceridemia (47.8%), which is correlated with anti-ds-DNA Ab, erythrocyturia, proteinuria, and SLEDAI/r index; decreased HDL cholesterol (28.4%) which significantly correlated with albumin, C3 and anti-ds-DNA Ab.

**Conclusions:**

In our patients with LN, MetS was associated with older age, impaired kidney function, and smoking. The most common parameter of MetS was arterial hypertension and dyslipidemia, which were significantly correlated with disease activity parameters, indicating an increased risk of cardiovascular complications in this group of patients.

## Abstract (sr)

**Uvod:**

Metabolički sindrom (MetS) kod bolesnika sa SLE, predstavlja dodatno opterećenje i loš prognostički faktor za nastanak ili pogoršanje ateroskleroze i za kardiovaskularne komplikacije. MetS je često inicijalno već ispoljen kod mnogih bolesnika sa lupus nefritisom (LN). Cilj našeg rada je bio da utvrdimo učestalost i karakteristike MetS kod bolesnika sa LN, kao i odnos komponenti MetS i aktivnosti lupus nefritisa.

**Metode:**

Kliničko ispitivanje je obuhvatilo grupu od 67 pacijenata sa LN, 54 (80,59%) ženskog pola i 13 (19,41%) muškaraca, prosečnih godina starosti 42,86±14,46. Pacijenti su podeljeni u dve grupe: prva sa MetS (35,82%) i druga bez MetS (64,18%), pacijenti su imali aktivan LN (34 ili 50,74%) i LN u remisiji (33 ili 49,25%). Pratili smo kliničke i biohemijske parametre od interesa.

**Rezultati:**

Poredeći pacijente sa LN zbirno kao i one sa MetS i bez MetS, utvrdili smo da su pacijenti sa MetS bili stariji (p=0,001), BMI (p<0,001) i sistolni pritisak je bio viši (p=0,002) i pušači su bili zastupljeniji u ovoj grupi (p<0,001). U analizama su zapaženi povišeni trigliceridi (p<0,001) i kreatinin (p=0,027) i snižen albumin (p=0,050) i GFR (p=0,020) u grupi sa MetS. MetS je bio zastupljen kod 44,11% pacijenata sa aktivnim LN i kod 27,7% sa LN u remisiji. Najzastupljeniji parameter MetS je bila arterijska hipertenzija (76,6%) koja značajno korelirala sa GFR i kreatininom; hipertrigliceridemija (47,8%) koja je korelirala sa anti-ds-DNA At, eritrociturijom, proteinurijom i SLEDAI/r indeksom; snižen HDL holesterol (28,4%) koji je korelirao značajno sa albuminom, C3 i anti-ds-DNA At.

**Zaključak:**

Kod naših pacijenata sa LN, MetS je bio povezan sa starijim životnim dobom, poremećajem bubrežne funkcije i pušenjem. Najzastupljeniji parameter MetS kod pacijenata sa LN je bila arterijska hipertenzija i dislipidemija koja je značajno korelirala sa parametrima aktivnosti bolesti, što upućuje na povećan rizik od kardiovaskularnih komplikacija u ovoj grupi bolesnika.

## Introduction

Systemic lupus erythematosus (SLE) is a very serious chronic immuno-inflammatory disease that ranks 20th on the list of causes of death in the female population [1]. An unfavorable outcome in SLE is most often caused by infections, malignancies, kidney lesions, and cardiovascular diseases [2]. Compared to the general population, patients with SLE have a 2- fold higher risk of developing nonfatal cardiovascular events, and compared to diabetics of the same age and gender, they have a 27% higher risk of developing cardiovascular disease [3]. The presence of metabolic syndrome (MetS) (obesity, arterial hypertension, hyperglycemia, hypertriglyceridemia, reduction of high-density lipoprotein cholesterol- HDL-cholesterol) in patients with SLE represents an additional burden and a poor prognostic factor for the onset or worsening of atherosclerosis and cardiovascular events. MetS has been described with a frequency of 16–44% in patients with SLE [4] [5] [6]. Patients with lupus nephritis (LN) represent a group in which the activity of SLE has led to kidney damage, which usually manifests clinically in the form of nephrotic, nephritic syndrome, or rapidly progressive glomerulonephritis with consequent arterial hypertension, all of which increase the risk of cardiovascular events. In patients with LN, MetS is often already present, which represents an additional risk for the development of accelerated atherosclerosis and cardiovascular complications [7] [8]. Patients with LN have a 47% higher chance of developing coronary artery disease compared to patients with SLE without LN [9].

Our work aimed to determine the frequency and characteristics of MetS in patients with LN, as well as the relationship components of MetS and characteristics of disease activity.

## Materials and methods

In the clinical examination (approved by the Ethics Committee and performed according to the tenets of the Declaration of Helsinki and conducted from 2012–2019), we included a group of 67 patients with SLE and LN (54–80.59% female and 13 – 19.41% male), the average age was 42.86±14.46 years. The diagnosis was confirmed by the criteria of the European League Against Rheumatism (EULAR), and LN was confirmed by kidney biopsy and pathohistological verification (WHO classification, and International Society of Nephrology/Renal Pathology Society (ISN/RPS) classification) [10] [11]. Kidney disease activity was also classified according to the renal disease activity index SLEDAI/r (renal (Systemic Lupus Erythematosus Disease Activity Index – rSLEDAI) [12]. SLEDAI/r consists of 4 criteria that grade renal impairment within the SLEDAI 2000 (Systemic Lupus Erythematosus Disease Activity Index- SLEDAI 2000) criteria of SLE activity [13]. The patients were divided into two groups: the first group consisted of patients with MetS (35.82%), and the second group consisted of patients without MetS (64.18%). Patients had active disease (34 patients – 50.74%) and disease in remission (33 patients – 49.25%). Active LN, according to standard analysis, was defined as proteinuria ≥0.5 g/24h; according to SLEDAI/r criteria (>4), hypocomplementemia C3 and C4, positive anti-double-stranded DNA antibodies (anti-ds-DNA Ab) and pathohistological findings of renal biopsy. Glomerular filtration rate (GFR) was defined according to the Chronic Kidney Disease Epidemiology Collaboration (CKDEPI) [14]. Complete remission was defined according to the criterion: proteinuria ≤0.5 g/24h.; SLEDAI/r index (<4), negative anti-ds-DNA antibodies, complement C3 and C4 within the reference range, and GFR ≥60 mL/min/1.73 m^2^). Excluding criteria were the same for all groups: patients with infection, positive urine culture, kidney failure (CKDeGFR< 60mil/min/ 1.73 m^2^), and those under 18.

MetS was defined as present if three or more of the following five criteria were present: (1) obesity (BMI >30 kg/m^2^ used as a surrogate marker of abdominal obesity consistent with the definition of abdominal obesity in the National Institutes of Health obesity guidelines; (2) elevated triglycerides ≥1.7 mmol/L, or medication; (3) reduced high-density lipoprotein (HDL) cholesterol (HDL-cholesterol) less than 40 mg/dL (1.04 mmol/L) in men, or less than 50 mg/dL (1.3 mmol/L) in women; (4); increased blood pressure ≥130/ 85 mmHg, or using medication for hypertension; (5) elevated fasting glucose (>5.6 mmol/L) or using antidiabetic medication [15].

All laboratory parameters for the group with active LN were determined before the immunosupressive treatment started (in this way, the effect of the therapy on the laboratory analyses was prevented). The group with LN in remission received maintenance therapy: 5–10 mg of corticosteroids and 50–75 mg of azathioprine per day. The authors had access to information that identified participants in the study.

Blood specimens were collected after an overnight fasting. The parameters we monitored were clinical parameters: BMI (kg/m^2^), arterial blood pressure (measured in millimeters of mercury: mmHg), standard laboratory parameters: C reactive protein (CRP), complete blood count (CBC), glucose, albumin, cholesterol, triglycerides, high-density lipoprotein cholesterol (HDL-cholesterol), low-density lipo protein cholesterol (LDL-cholesterol), and kidney function parameters: serum creatinine levels and GFR. Regarding immune parameters, complement C3 (C3) and complement C4 (C4), anti-antinuclear antibodies (ANA), and anti-double stranded DNA antibodies (anti-ds-DNA Ab) were monitored. Complete urine analysis for urinary casts, erythrocyturia, pyuria, and proteinuria 24-hour and urine culture were monitored.

### Statistical analysis

The data were analysed using the Statistical Package for the Social Sciences IBM-SPSS, version 26.0. Categorical variables were presented as frequency and were analysed using the Chi-square test. All continuous variables are presented as median (interquartile range: 25–75th percentile) or mean± standard deviation for the data that are not normally or normally distributed, respectively. The Kolmogorov-Smirnov test was used to test the normality of data distribution. For intergroup comparisons, the Mann-Whitney test for non-parametric variables was used. Spearmen’s coefficient correlation tested the relationship between variables. Statistical significance was defined as p<0.05 for all comparisons.

## Results

Basic demographic, clinical, and laboratory parameters of patients with LN, as well as with and without MetS, are shown in Table 1.

**Table 1:** Basic characteristics of our patients with LN collectively and divided according to the presence of MetS. * Chi-square test; # Mann-Whitney test (bold values are significant) <br>MetS – metabolical syndrome; nMetS – non-metabolical syndrome

Parameters	LN 67	MetS 24	nMetS 43	p-value
Sex: male/ female N, %	13 (19.4)/54 (80.6)	8 (33.3)/16 (66.7)	5 (11.6)/38 (88.4)	0.031*
Age (years)	41 (31–55)	54 (43–59)	36 (30–43)	**0.001#**
BMI (kg/m^2^)	24.6 (22.9–28.6)	29.75 (25.22–32.92)	23.40 (21.50–24.80)	**<0.001#**
Systolic blood pressure	130 (120–135)	135 (130–140)	120 (110–130)	**0.002#**
Diastolic blood pressure	80 (70–85)	80 (72.5–90.0)	80 (70–85)	0.181#
Smoking	20 (29.8)	14 (58.3)	6 (14.0)	<0.001*
CRP (mg/L)	3.45 (3.10–4.90)	3.45 (3.11–4.77)	3.45 (2.97–5.10)	0.699#
RBC (10^9^ g/L)	4.12 (3.70–4.55)	3.93 (3.51–4.59)	4.15 (3.94–4.53)	0.224#
Hb (g/L)	118 (107–126)	115.50 (98.50–130.50)	120 (112–125)	0.534#
WBC (10^9^ g/L)	6.24 (4.87–7.94)	6.55 (5.19–7.88)	5.52 (4.43–8.05)	0.221#
PLT (10^9^ g/L)	202 (178–254)	206.50 (174.25–252.00)	199 (180–256)	0.880#
Triglyceride (mmol/L)	1.62 (1.32–2.11)	1.94 (1.73–2.73)	1.48 (1.20–1.64)	**<0.001#**
Cholesterole (mmol/L)	5.61 (4.80–6.51)	6.25 (4.43–6.91)	5.60 (4.88–6.23)	0.855#
HDL-cholesterol (mmol/L)	1.70 (1.40–1.96)	1.62 (1.05–1.91)	1.80 (1.50–1.99)	0.131#
LDL cholesterol (mmol/L)	3.50 (2.81–4.12)	3.74 (2.50–4.20)	3.50 (2.89–3.99)	0.917#
Glucose (mmol/L)	4.60 (4.40–5.20)	5.10 (4.40–5.77)	4.60 (4.40–5.00)	0.067#
Creatinine (μmol/L)	79 (67–109)	95.50 (72.25–123.25)	77.00 (64.00–94.00)	**0.027#**
Albumin (g/L)	38 (32–41)	34.00 (28.25–40.00)	39.00 (35.00–41.00)	**0.050#**
GFR (mL/min/1,73 m^2^)	78.42 (60.10–102.51)	66.30 (51.15–89.91)	83.01 (68.90–109.76)	**0.020#**
Proteinuria (g/24h)	1.04 (0.25–3.60)	1.68 (0.37–6.00)	0.41 (0.24–3.20)	0.055#
SLEDAI/r	1 (0–6)	3.50 (0–6.75)	1 (0–5)	0.093#
C3 complement (g/L)	0.81 (0.65–0.90)	0.76 (0.63–0.97)	0.81 (0.65–0.85)	0.979#
C4 complement (g/L)	0.13 (0.09–0.17)	0.14 (0.11–0.17)	0.12 (0.08–0.17)	0.192#
ANA (IU/mL)	2 (0–3)	2.00 (1.00–3.00)	1.00 (0.00–13.00)	0.968#
Anti ds DNA Ab (IU/mL)	40 (15–100)	47.50 (20.00–100.00)	15.00 (15.00–100.00)	0.195#

Comparing patients with LN collectively as well as those with and without MetS, we found statistical significance for age. Patients with MetS were older (p=0.001). BMI was significantly higher (p<0.001) in the group with MetS; the average BMI was 29.89±4.85 kg/m^2^ (in the group without MetS 23.35±2.70 kg/m^2^), while for the collective group of patients, it was 24.69±4.77 kg/m^2^. A statistically significant difference was obtained with smokers in the group with MetS (p<0.001). Also, in the group with MetS, systolic blood pressure was statistically significantly elevated (p=0.002). Comparing laboratory analyses, a statistically significant difference was observed for triglycerides (p<0.001), albumin (p=0.050), and renal function parameters: creatinine (p=0.027) and GFR (p=0.020). Proteinuria was increased in the group with MetS, but in the comparison, a value at the limit of significance was obtained (p=0.055). MetS was present in 15 (44.11%) patients with active LN and in patients with LN in remission in 9 (27.27%). Table 2 and Table 3 show basic clinical and laboratory parameters in patients with active LN and LN in remission.

**Table 2:** Characteristic clinical and laboratory parameters of patients with active LN according to the prevalence of MetS. Mann-Whitney test (bold values are significant) <br>MetS – metabolical syndrome; nMetS – non-metabolical syndrome

LN – active			
Parameters	MetS (15/34)	nMetS (19/34)	p
BMI (kg/m^2^)	29.80 (24.60–33.50)	23.20 (21.50–24.70)	**<0.001**
Glucose (mmol/L)	5.10 (4.30–5.80)	4.60 (4.40–4.80)	0.302
Triglyceride (mmol/L)	2.29 (1.72–3.11)	1.50 (1.30–1.70)	**0.001**
HDL-cholesterol (mmol/L)	1.69 (1.04–1.96)	1.70 (1.50–1.96)	0.336
Systolic blood pressure (mmHg)	135 (130–140)	120 (110–130)	**0.027**
Diastolic blood pressure (mmHg)	80 (80–90)	80 (70–80)	0.202
CRP (mg/L)	3.48 (2.97–5.29)	3.47 (0.96–5.80)	0.732
Creatinine (μmol/L)	111 (70–137)	71 (64–87)	0.096
C3 (g/L)	0.72 (0.45–0.77)	0.72 (0.52–0.76)	0.973
C4 (g/L)	0.13 (0.10–0.19)	0.08 (0.05–0.12)	**0.043**
Proteinuria (g/24h)	3.72 (1.90–9.70)	3.20 (1.75–4.10)	0.179
SLEDAI/r	6 (4–7)	6 (3–6)	0.179

**Table 3:** Characteristic clinical and laboratory parameters of patients with LN in remission according to the presence of MetS. Mann-Whitney test (bold values are significant) <br>MetS – metabolical syndrome; nMetS – non-metabolical syndrome

LN – in remission			
Parameters	MetS (9/33)	nMetS ( 24/33)	p
BMI (kg/m2)	29.60 (25.60–32.30)	23.60 (21.60–26.22)	**<0.001**
Glucose (mmol/L)	5.20 (4.40–6.60)	4.65 (4.32–5.00)	0.121
Triglyceride (mmol/L)	1.82 (1.73–2.25)	1.39 (1.09–1.61)	**0.016**
HDL-cholesterol (mmol/L)	1.60 (1.23–1.87)	1.80 (1.50–2.08)	0.272
Systolic blood pressure (mmHg)	135 (130–140)	120 (116.25–133.75)	**0.029**
Diastolic blood pressure (mmHg)	80 (70–90)	80 (70–85)	0.592
CRP (mg/L)	3.20 (3.13–4.10)	3.43 (3.00–4.25)	0.921
Creatinine (μmol/L)	79 (73.5–110.5)	78.50 (64.75–102.25)	0.414
C3 (g/L)	0.98 (0.87–1.08)	0.84 (0.81–1.00)	0.207
C4 (g/L)	0.15 (0.13–0.16)	0.14 (0.12–0.19)	0.921
Proteinuria (g/24h)	0.28 (0.14–0.78)	0.25 (0.15–0.36)	0.677
SLEDAI/ r	0 (0–0.5)	0 (0–1)	0.766

We obtained a statistically significant difference for MetS parameters (BMI, triglycerides, systolic blood pressure) in LN with active disease and LN in remission. Table 4 shows the prevalence of certain MetS parameters in our patients.

**Table 4:** The frequency of certain parameters of MetS in the group with LN.

LN (n=67)	
MetS, n (%)	24 (35.8)
**BMI>30, n (%)**	14 (20.9)
BMI, median (IQR)	24.60 (22.90–28.60)
**Hypertriglyceridiemia, n (%)**	32 (47.8)
Triglyceride, median (IQR)	1.62 (1.32–2.11)
**Low HDL-cholesterol, n (%)**	19 (28.4)
HDL-cholesterol, median (IQR)	1.70 (1.40–1.96)
**Arterial hypertension, n (%)**	50 (74.6)
Systolic blood pressure, median <br>(IQR)	130 (120–135)
Diastolic blood pressure, median <br>(IQR)	80 (70–85)
**Hyperglycemia, n (%)**	9 (13.4)
Glucose, median (IQR)	4.60 (4.40–5.20)

In Table 4, we have shown the prevalence of certain MetS parameters in our patients with LN. The most prevalent was arterial hypertension (74.6%), dyslipidemia: increased triglycerides (47.8%), decreased HDL-cholesterol (28.4%), BMI in 20.9%, and hyperglycemia was observed in 13.4% of patients.

MetS correlated with parameters of renal function: GFR and creatinine and with albumin, erythrocyturia, and borderline significance for proteinuria (p=0.054) were also noted, as shown in Table 5. If we look at the correlations between individual elements of MetS with parameters that are significant for disease activity in our patients with LN, we note that BMI correlates significantly with GFR, creatinine, and erythrocyturia. The level of serum triglycerides correlates statistically significantly with anti-ds-DNA Ab, and urinary parameters: erythrocyturia, proteinuria, and SLEDAI/r index, and HDL cholesterol with albumins, complement C3, and anti-ds-DNA Ab. Arterial hypertension correlates significantly with renal function parameters GFR and creatinine.

**Table 5:** Correlation of MetS and significant LN parameters. Spearman’s correlation rank (bold values are significant)

		MetS	GFR	Creatinine	Albumin	C3	ANA	ds DNA <br>Ab	Er/u	Protein/ <br>u 24h	SLEDAI/r
MetS	r	1.000	**-0.286**	**0.273**	**-0.241**	-0.00,	0.005	0.162	**0.258**	0.237	0.206
	p		**0.019**	**0.025**	**0.049**	0.979	0.968	0.197	**0.035**	0.054	0.094
BMI	r	**0.656**	**-0.307**	**0.281**	-0.099	0.025	-0.042	0.019	**0.251**	0.065	0.072
	p	**0.000**	**0.011**	**0.021**	0.426	0.839	0.734	0.879	**0.040**	0.603	0.564
Trygliceride	r	**0.533**	-0.156	0.195	-0.233	-0.211	0.111	**0.256**	**0.295**	**0.308**	**0.253**
	p	**0.000**	0.208	0.144	0.058	0.086	0.370	**0.040**	**0.015**	**0.011**	**0.039**
HDL-cholesterol	r	-0.186	-0.115	0.041	**0.334**	**0.290**	-0.111	**-0.246**	-0.068	-0.223	-0.168
	p	0.132	0.355	0.740	**0.006**	**0.017**	0.371	**0.048**	0.583	0.070	0.174
Glucose	r	0.225	-0.206	0.184	-0.041	0.033	0.040	-0.028	0.077	0.013	0.022
	p	0.067	0.095	0.136	0.741	0.793	0.749	0.828	0.538	0.916	0.860
Arter. <br>hypertension	r	**0.247**	**-0.250**	**0.280**	-0.217	0.012	-0.003	-0.011	0.064	0.124	0.073
	p	**0.044**	**0.041**	**0.022**	0.077	0.922	0.984	0.928	0.605	0.319	0.558

## Discussion

MetS and LN, as the renal manifestation of SLE, represent two causally related entities. Patients with active LN have a higher frequency of MetS than those with LN in remission. MetS further aggravates LN with its components, damaging many organs and systems. Previous studies mainly described patients with SLE as a target group and indicated the severity of MetS, while much fewer described the association between MetS and LN [7] [8] [9].

Patients with SLE and LN have accelerated development of atherosclerosis, which is conditioned by the action of traditional risk factors (smoking, dyslipidemia, arterial hypertension, diabetes mellitus, increased BMI) and the immuno-inflammatory effect of the disease, while the mechanism of this process has not yet been fully clarified [16] [12]. The treatment of SLE involves not only the treatment of the immune process but also the prevention of cardiovascular complications and atherosclerosis. According to many findings, endothelial dysfunction is the basis of the development of atherosclerosis, and SLE is an independent factor of endothelial dysfunction [17] [18]. The results of a multicenter study by Parker et al., [8]. which included 1150 patients with SLE, describe a prevalence of MetS of 38.2% and also indicate that lupus nephritis and active disease are significant factors in the development of MetS, where the early use of antimalarials had a protective effect. A meta-analysis by Lu et al. [19], which included the results of 20 studies, shows that patients with LN have twice the risk of developing cardiovascular diseases (atherosclerosis, myocardial infarction, stroke, peripheral vascular disease, heart failure) compared to patients with SLE (who have a 2–10 times higher risk compared to the general population).

Sharma et al. investigated subclinical atherosclerosis in patients with SLE and LN by measuring the thickening of the intima-media of the carotid artery complex and showed that patients with LN have a higher risk of developing atherosclerotic changes in the carotid arteries [20].

In our group of patients with LN, MetS was observed in a similar percentage (35.82%) as in other authors [8]. We observed that in 44.1% of patients with active LN, MetS was manifested initially, while in patients with LN in remission, MetS was observed in 27.2% of cases. According to the age distribution, patients with LN and MetS were significantly older, and, quite expectedly, BMI, arterial hypertension, and smoking were significantly more prevalent in this group.

The prevalence of obesity in SLE was 28–50% and was associated with dyslipidemia and atherosclerosis, with older age, lupus nephritis, arterial hypertension, duration of the underlying disease, and corticosteroid therapy [21] [22]. Obesity was statistically significantly associated with the development of LN during the follow-up of patients with SLE, according to a study by Kang et al. [23]. Elevated BMI in our patients with LN was represented in a slightly lower percentage of 20.9% for BMI >30 kg/m^2^, and 41.7% for BMI 25–29 kg/m^2^. Sun et al. described arterial hypertension in 91% of patients with LN, where the average values of systolic blood pressure of their patients were 130.7±15.5 mmHg and diastolic blood pressure 78.2±10.3 mmHg [9]. Arterial hypertension, as an important predictor of adverse cardiovascular outcomes in the general population, was present in 91.6% of our patients with LN and MetS, and in the total number of patients with LN, arterial hypertension was present in 74.6% of patients.

In our patients with LN, the systolic blood pressure was higher than that described by other authors and was 140.93±11.42 mmHg, as well as the diastolic blood pressure (84.61±7.40 mmHg). These elevated arterial pressure values were associated with active kidney lesions in LN and elevated immune parameters. Patients with SLE had arterial hypertension in 58.2% and obesity in 12.4%, according to the study by Katz et al. [24], and a similar percentage of 58.4% was described by Hanly et al. [25] in an international cohort study [25], in contrast to our patients, who had a higher percentage of arterial hypertension.

The difference between the subjects explains such a high percentage of arterial hypertension in our patients. Katz et al. [24] described a group of patients with SLE, while our group was represented by patients with LN, which was the most severe manifestation of SLE. In the study by Hanly et al., a group of patients with LN was described who had elevated BMI on average 5.9% of patients, in contrast to ours (20.9%), which resulted in higher arterial hypertension.

One of the important characteristics of the MetS in SLE is atherogenic dyslipidemia, most often reduced HDL-cholesterol, elevated triglycerides, and LDL-cholesterol is usually within reference limits. It is considered that the role of HDL-cholesterol in SLE is very important from the aspect of the anti-inflammatory response, and some studies have shown that the infusion of HDL-cholesterol reduces atherosclerotic plaque in an animal model [26] [27]. Sun et al. [9] describe dyslipidemia in LN in 45.5% of patients. Dyslipidemia is also very common in our patients: hypertriglyceridemia 47.8%, hypercholesterolemia 59.7%, and lowered HDL-cholesterol (28.4%). Our patients with LN also had elevated LDL cholesterol, which further increased the risk of developing cardiovascular complications.

Lin et al. indicate that patients with newly diagnosed SLE have a 22% higher risk compared to the control group for the development of diabetes mellitus in the next three years, whereby insulin resistance is influenced by diseases and applied therapy (corticosteroids, calcineurin inhibitors), genetic factors, race and other factors [28]. All this indicates that in patients with SLE, regular controls are necessary – fasting plasma glucose level and glycated hemoglobin, control of the therapeutic level of calcineurin inhibitors in the blood, reduction of the maintenance dose of corticosteroids, as well as adherence to a dietary regime of nutrition.

A study by Salmasi et al. [29], which included 1498 patients with SLE, showed a protective effect of antimalarial drugs that reduced the risk of diabetes mellitus in these patients by 39%, mainly by reducing the need for high doses of corticosteroids at the same time.

Our patients had manifested hyperglycemia in 13.4% of cases, which is a lower percentage than that described by other authors [29] [30]. We believe many factors contributed to this: our patients were younger, their BMI was lower, and they might have been more careful with the recommended diet.

Many studies indicate an association between renal function parameters and MetS, i.e., an increased cardiovascular risk that is accompanied by elevated creatinine and MetS. In a study by Bultinik et al. [31] multivariate analysis of the metabolic syndrome score and clinical and therapeutic variables was used to obtain statistical significance for age, ESR, complement C3, creatinine, and intravenous corticosteroid therapy. These authors note that corticosteroid therapy affects the worsening of arterial hypertension, dyslipidemia, obesity in patients with SLE, and especially in patients with lupus nephritis and lupus CNS, as the most severe manifestations of SLE, bearing in mind that intravenous doses of corticosteroids are applied much more often [31].

In our patients, we observe a statistically significant correlation of MetS with kidney function parameters (creatinine and GFR), but also with albumin and erythrocyturia, which is similar to the results of other studies [31].

Kidney failure, which represents poor prognostic parameters in LN, was more pronounced in the group of patients with active LN and MetS. It confirms that this is a group of patients with an increased risk for a poor outcome. A decrease in serum albumin and manifested erythrocyturia were also observed, which are important parameters for LN activity, and their significant correlation with MetS indicates the connection of these unfavorable factors and the necessity of careful monitoring of these patients and implementation of timely treatment.

Also, when correlating the individual parameters of MetS with parameters significant for LN activity, we notice that dyslipidemia (elevated triglycerides and decreased HDL-cholesterol level) significantly correlates with many parameters for disease activity. Triglycerides are statistically significantly correlated with anti-ds-DNA Ab and urinary parameters: erythrocyturia, proteinuria, SLEDAI/r index, and HDL-cholesterol correlated with albumin, C3, and anti-ds- DNA Ab. In our group, arterial hypertension as a parameter of MetS shows a significant correlation with parameters of renal function: creatinine and GFR.

The results of our study indicate the necessity of a multidisciplinary approach to patients with LN, so in addition to achieving remission of glomerulonephritis, we could also influence other adverse conditions (MetS) and ensure a favorable course of the disease and better survival of our patients.

However, in our opinion, a limitation of our study was related to the presented number of patients who had LN and MetS. The sample size most likely limited the statistical significance of the correlation between MetS elements and certain parameters of active renal lesions (complement level, proteinuria, SLEDAI/r index), but individual MetS parameters (triglyceride and HDL cholesterol levels) showed that significance. This is also why we believe that future studies with more patients with active LN and manifested MetS could better define this relationship.

## Conclusions

The presence of MetS in our LN patients was associated with age, more severe impairment of renal function, and smoking. The most common parameters of MetS were arterial hypertension and dyslipidemia, which are significantly correlated with disease activity parameters, indicating an increased risk of cardiovascular complications in this group of patients.

This is also the reason to highlight the need for timely detection and treatment of MetS components in patients with LN. Bearing in mind that LN activity and cardiovascular complications are very significant predictors of an unfavorable outcome, detailed follow- up of these patients could influence a more favorable course and delay the progression of the disease.

## Dodatak

### Statement of ethics

Our institution’s Ethics Committee approved the study, and oral informed consent was obtained from all patients.

### Conflict of interest statement

All the authors declare that they have no conflict of interest in this work.
